# SERS and Indicator Paper Sensing of Hydrogen Peroxide Using Au@Ag Nanorods

**DOI:** 10.3390/s22093202

**Published:** 2022-04-21

**Authors:** Boris N. Khlebtsov, Andrey M. Burov, Andrey M. Zakharevich, Nikolai G. Khlebtsov

**Affiliations:** 1 Institute of Biochemistry and Physiology of Plants and Microorganisms, Saratov Scientific Centre of the Russian Academy of Sciences (IBPPM RAS), 410049 Saratov, Russia; burov_a@ibppm.ru (A.M.B.); khlebtsov_n@ibppm.ru (N.G.K.); 2 Department of Physics, Saratov State University, 410012 Saratov, Russia; lab-15@mail.ru

**Keywords:** hydrogen peroxide, Au@Ag nanorods, etching, SERS

## Abstract

The detection of hydrogen peroxide and the control of its concentration are important tasks in the biological and chemical sciences. In this paper, we developed a simple and quantitative method for the non-enzymatic detection of H_2_O_2_ based on the selective etching of Au@Ag nanorods with embedded Raman active molecules. The transfer of electrons between silver atoms and hydrogen peroxide enhances the oxidation reaction, and the Ag shell around the Au nanorod gradually dissolves. This leads to a change in the color of the nanoparticle colloid, a shift in LSPR, and a decrease in the SERS response from molecules embedded between the Au core and Ag shell. In our study, we compared the sensitivity of these readouts for nanoparticles with different Ag shell morphology. We found that triangle core–shell nanoparticles exhibited the highest sensitivity, with a detection limit of 10^−4^ M, and the SERS detection range of 1 × 10^−4^ to 2 × 10^−2^ M. In addition, a colorimetric strategy was applied to fabricate a simple indicator paper sensor for fast detection of hydrogen peroxide in liquids. In this case, the concentration of hydrogen peroxide was qualitatively determined by the change in the color of the nanoparticles deposited on the nitrocellulose membrane.

## 1. Introduction

Hydrogen peroxide is the simplest representative of peroxides, which is of great importance for bioanalysis [1], cell biology [2,3], and chemical sciences [4,5]. Simple and accurate determination of H_2_O_2_ concentration in the micromolar range is very important for all these technologies. As a rule, methods based on enzymes (for example, peroxidase) are used in combination with colorimetric [6,7] or fluorescence [8,9] detection. At the same time, several methods have recently been proposed using nanoparticles as sensors with peroxidase activity [10,11]. However, the impurities adsorb to the surface of the nanoparticles strongly reduce the catalytic activity, which limits their applications. Another possible approach for the detection of hydrogen peroxide is based on the unique optical properties of metal nanoparticles. Au and Ag nanoparticles show a localized surface plasmon resonance—strong light absorption and scattering in the VIS–NIR region related to an oscillation of the conduction electrons. For sensing applications, the intensity of the LSPR peak should depend strongly on the size, shape, and environment of the nanoparticles [12]. Based on this, many optical biosensors have been designed to detect cancer biomarkers [13], pathogens [14], and metal ions and biomolecules [15].

H_2_O_2_ can oxidize Ag (E_0_ = 0.7996 V) both in acidic (E_0_ = 1.7736 V) and basic (E_0_ = 0.867 V) conditions [16]. Thus, when H_2_O_2_ is added to Ag nanoparticles, they can be etched to silver ions, which leads to a transformation in shape and size and can be detected by changing the position and intensity of LSPR. This is the basis of numerous colorimetric plasmon sensors for quantitative analysis [17,18,19] with the advantages of low cost, relatively high sensitivity, and absence of interference with biomolecules. AgNPs with different shapes have been used for the detection of H_2_O_2_ including triangular [16], spherical [18,20], core–shell [21,22,23], cubic [24], and many others [24,25,26]. It is now believed that small particles (for example 5 nm nanoclusters) and nanoparticles with sharp tips and edges (for example, triangle prisms) demonstrate higher sensitivity to H_2_O_2_, compared with spherical particles [24]. The increased sensitivity of non-spherical particles is due to the greater chemical reactivity of sharp edges and the greater shift in plasmon resonance with changes in shape than particle size [24]. On the other hand, sharp edges can be easily etched by hydrogen peroxide as well as by other molecules, metals, and ions [27], which reduces the sensing specificity. Bimetallic (Au@Ag, Au@Pd/Au @Pt, and Pt@Pd) have advantages over monometallic due to greater stability and the ability to combine unique optical and catalytic properties [28,29,30]. The main method for reading the signal is colorimetry (change in optical density) [20,22,23], although there are also complex methods based on changing fluorescence [25] or SERS [21,24,31].

Au@Ag core–shell nanorods with embedded Raman reporters [32] are promising labels for biosensing because of several important advantages related to monodispersity, sharp-contrast multicolor change by Ag shell tuning, the possibility to produce nanoparticles with different shapes, and outstanding SERS response. The protocol for the synthesis of such particles consists of three main steps [33]. First, gold nanorods are synthesized. The second step is the functionalization of the surface of the rods with Raman reporters (usually thiolated aromatic molecules). Finally, a layer of silver is additionally restored on the surface of the functionalized nanorods. As a result, core–shell nanorods are obtained, which have the morphology of a cuboid [33], nanospindle [34], octadecahedron [35], etc., depending on the molar ratio of Au and Ag. During the growth of the silver shell, an increase in the optical density of the colloid, a change in color, and a dramatic increase in SERS are observed. The unusually high sensitivity of changes to the thickness of the silver shell should be noted. Indeed, even an increase in the shell thickness by only 1 nm leads to a shift in the plasmon resonance by 50 nm and an increase in SERS by a factor of 5 [32]. We assume that a decrease in the thickness of the shell during its etching with hydrogen peroxide should also lead to large changes in the spectrum and SERS, which can be used to quantify the concentration of H_2_O_2_. In a recent paper, the sensitivity of the shift in the plasmon resonance of nanorods to a micromolar concentration of hydrogen peroxide has already been demonstrated [36].

In this study, we investigated the sensitivities of colorimetric, spectral, and SERS changes upon hydrogen peroxide etching of Au@Ag nanorods with embedded Raman reporters. Two variants of sensors were considered: the traditional colloid-based approach and a paper-based format obtained by applying particles to a nitrocellulose membrane.

## 2. Materials and Methods

### 2.1. Reagents and Materials

Cetyltrimethylammonium bromide (CTAB, >98.0%), cetyltrimethylammonium chloride (CTAC, 25% water solution), hydroquinone (HQ, 99%), L-ascorbic acid (AA, >99.9%), 4-nitrobenzenethiol (NBT), Hydrogen peroxide (37%), and sodium borohydride (NaBH_4_, 99%) were purchased from Sigma-Aldrich. Hydrogen tetrachloroaurate trihydrate (HAuCl_4_·3H_2_O) and silver nitrate (AgNO_3_, >99%) were purchased from Alfa Aesar. Ultrapure water obtained from a Milli-Q Integral 5 system was used in all experiments.

### 2.2. Synthesis of Au@Ag Nanorods with Embedded NBT

**Figure 1:**
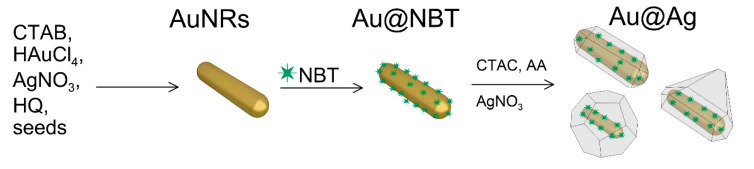
Scheme for the synthesis of Au@Ag nanorods with embedded Raman reporters.

Au@Ag nanorods with embedded Raman reporters were obtained by using a three-stage protocol, schematically represented in Figure 1. First, Au nanorods (AuNRs) were firstly prepared by a seed-mediated growth, as described by Vigderman and Zubarev [37], and resuspended in 10 mM CTAC solution with an Au concentration of 0.5 mM. In the second step, 300 µL of 2 mM NBT solution in ethanol was added to 10 mL AuNRs, followed by incubation at room temperature for 1 h. To wash all unbound reporter molecules, the nanoparticles were centrifuged three times at 9000× *g* for 10 min and redispersed in 20 mM CTAC solution. In the third step, 4 mL of NBT functionalized nanorods (Au concentration 0.5 mM) were mixed with 12 mL of 20 mM CTAC and various amounts (32, 64, 128, 256, 512, 1024 µL) of 10 mM AgNO3 solution, followed by the addition of 4 time excess (128, 256, 512, 1024, 2048, 4096 µL) of 10 mM ascorbic acid. The mixture was incubated at 70 °C for 3 h.

Resulted core–shell particles with embedded NBT were centrifuged at 7000× *g* for 10 min and resuspended in 16 mL of water. Thus, we obtained 6 samples of Au@Ag nanorods with different Ag/Au molar ratios.

### 2.3. Nanoparticles Characterization

Extinction spectra were measured with a Specord 300 spectrophotometer (Analytik, Jena, Germany). Transmission electron microscopy (TEM) images were recorded on a Libra-120 transmission electron microscope (Carl Zeiss, Jena, Germany) at the Simbioz Center for the Collective Use of Research Equipment in the Field of Physico–Chemical Biology and Nanobiotechnology, IBPPM RAS, Saratov. SEM images of nanoparticles were made using MIRA TESCAN II scanning electron microscope. SERS spectra were acquired with a Peak Seeker Pro 785 Raman spectrometer (Ocean Optics) in 1 cm quartz cuvettes using 785 nm irradiation (30 mW). The acquisition interval was 10 s and all spectra were averaged over 3 independent runs.

### 2.4. Detection of H_2_O_2_ in Colloid

For this study, core–shell particles with molar ratios of silver to gold equal to 1.25, 2.5, and 5 were chosen. Each colloid was divided into 10 tubes of 1 mL each. Various amounts of H_2_O_2_ were added to 1 mL of nanoparticle colloid to have a final hydrogen peroxide concentration ranging from 2 µM to 50 mM. After 1 h of incubation at room temperature colloids were tested by visual inspection, extinction spectra measurement, and SERS measurement.

### 2.5. Indicator Paper for the Detection of H_2_O_2_

A nitrocellulose membrane (MDI, India) was used to prepare indicator paper. Briefly, 1 µL of nanoparticles were applied to the membrane surface. After drying for 1 h, the nanoparticles formed spots with a color corresponding to the color of the colloid. The indicator paper was placed in a peroxide solution with a weighed concentration (from 0.5 to 50 mM) for 1 min. Next, the indicator paper was dried in an oven at 40 °C for 10 min. Detection of hydrogen peroxide was carried out visually by changing the color of the spots. For the detection of peroxide in milk samples, a product obtained from a local store was used. The sample was artificially contaminated with hydrogen peroxide, and testing using indicator paper was carried out as described above.

## 3. Results

### 3.1. Size, Shape, and SERS Characterization of the Synthesized Nanoparticles

The core–shell particles used in the study were obtained using a three-stage scheme [33]. In the first stage, gold nanorods (AuNRs) were obtained. For optimal SERS detection, the plasmon resonance of the nanorods should be in the NIR region. To this end, we used a well-known seed-mediated protocol developed by Vigderman and Zubarev [37]. The obtained AuNRs were 12 ± 3 nm in diameter and 62 ± 8 nm in length (Figure S1). The nanorods had an average axial ratio of 5.2, which is in good agreement with the wavelength positions of the dipolar longitudinal (920 nm) and transversal (507 nm) plasmon resonances (Figure S1, Supporting information). The position of the transversal mode corresponded to the typical orange-brown color of the colloid. In the second stage, the AuNRs were functionalized with NBT via covalent bonding. NBT was chosen because of its high Raman cross-section and the unique chemical structure that promotes the growth of an anisotropic silver shell [34]. The functionalization of the AuNRs surface by Raman reporters resulted in a minor, few-nanometer redshift of the longitudinal plasmon resonance. At the last stage of synthesis, the silver shell was grown on the surface of functionalized nanorods. We studied different molar ratios of silver to gold from 0.15 to 5. Growth of the silver shell led to notable color changes in colloids depending on the amount of reduced Ag. These color changes covered orange-brown (for original nanorod colloid), yellow-green, green, blue, red, and finally bright yellow (Figure S2, Supporting Information). Previously, it was reported that silver atoms are deposited mainly on the side faces of a gold nanorod [35]. This leads to a decrease in the axial ratio of particles and, together with the plasmonic properties of Ag, shifts the plasmon resonance to the visible part of the spectrum. It should be noted that the use of NBT-functionalized nanorods produces an anisotropic Ag shell. The final shape of the particles can be a spindle-shaped, triangular prism, or octahedral while using CTAB-coated nanorods lead to the formation of cuboids [33]. Another obvious consequence of the growth of the silver shell is an increase in the optical density of the colloid due to more metal in the sample.

**Figure 2:**
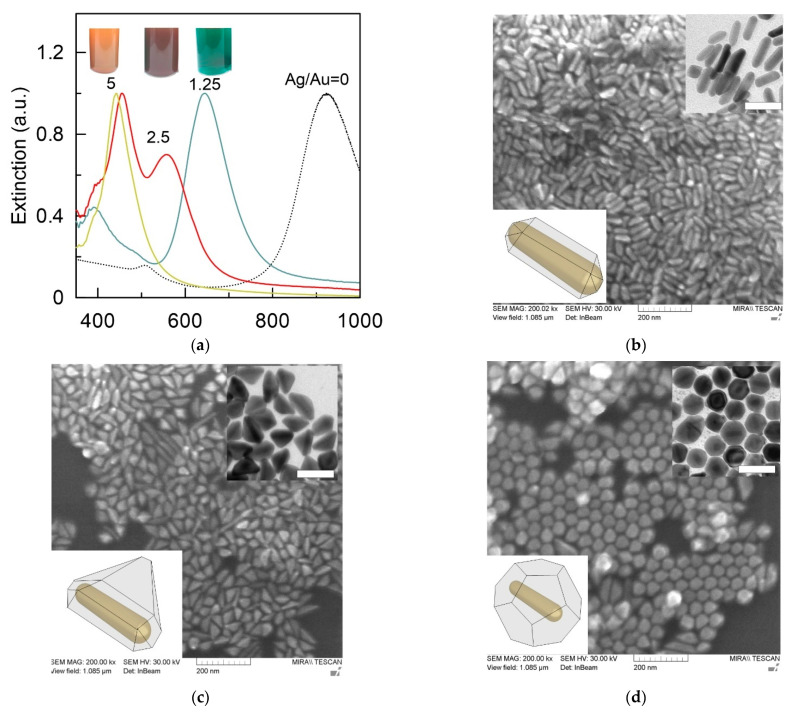
(**a**) Extinction spectra of Au@Ag core–shell nanorods with embedded NBT. The nanoparticles were obtained using Ag-to-Au molar ratios 5 (yellow curve), 2.5 (red curve), and 1.25 (green curve). The dashed curve corresponds to extinction spectra of the original AuNRs The insert show image of bottles with Au@Ag core–shell nanorods (b-в) SEM image of Au@Ag core–shell nanorods obtained using Ag-to-Au molar ratios of 1.25 (**b**), 2.5 (**c**), and 5 (**d**). The insert on the top corresponds to the TEM image of the nanoparticles. The scale bar is 100 nm. The left-bottom inserts show the most probable structure of nanoparticles.

Figure 2a shows the normalized extinction spectra of Au@Ag nanorods with embedded NBT obtained at silver to gold molar ratios of 1.25, 2.5, and 5. For comparison, the dashed curve shows the normalized extinction spectrum of the initial gold nanorods. It can be seen that, as the silver content increased, the plasmon resonance shifted progressively from 920 nm to 650, 550, and 450 nm, which led to a change in the color of the colloid to green, red, and bright yellow, respectively. Notably, the shift in the plasmon resonance was also accompanied by an increase in extinction in the wavelength range of 400–450 nm, which is characteristic of silver colloids. Next, we studied the size and shape of the resulting particles. At a relatively low Ag/Au ratio (Figure 1b), nanoparticles had the shape of core–shell nanorods with a slight asymmetric displacement of the gold nanorod inside the silver shell (insert in Figure 2b). The average particle thickness according to TEM data increased to 16 ± 4 nm, while the length remained almost unchanged at 63 ± 8 nm. This once again confirmed the adsorption of silver to predominantly lateral faces of the gold nanorod during shell growth. With an increase in the Ag/Au ratio to 2.5, further growth of the silver shell along one of the faces was observed, which led to the formation of particles with a predominant triangular pyramid morphology (Figure 2c). Finally, with the largest amount of added silver, the final nanoparticles were a silver octadecahedron with a gold nanorod inside (Figure 2d). It should be noted that the influence of the nanorod on the optical properties of such particles was minimal. Thus, only one maximum near 450 nm was observed in the extinction spectrum (Figure 2a), which is characteristic of silver particles, with a size of about 70 nm.

**Figure 3:**
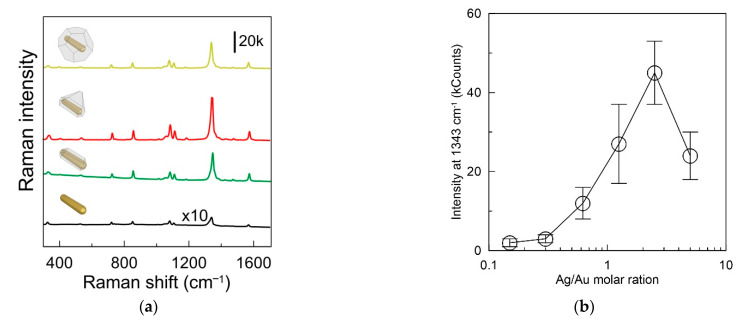
(**a**) SERS spectra of Au@Ag core–shell nanorods with embedded NBT. The nanoparticles were obtained using Ag-to-Au molar ratios 5 (yellow curve), 2.5 (red curve), and 1.25 (green curve). The black curve corresponds to SERS spectra of the AuNRs functionalized with NBT; (**b**) the dependence of SERS intensity of NO_2_ vibration on the Ag/Au molar ratio.

Let us discuss further the SERS properties of NBT functionalized AuNRs and core–shell nanoparticles with embedded Raman reporters. SERS spectrum of NBT on AuNR is shown in Figure 3a (black curve). In agreement with previous findings [38], the SERS spectrum is dominated with major ν (NO_2_) peak located at 1343 cm^−1^ and several minor Raman bands associated with the δ (CS) at 390 cm^−1^, γ (CCC) at 560 cm^−1^, π (CH) at 854 cm^−1^, ν (CS) at 1081 cm^−1^, and ν (CC) at 1569 cm^−1^. In the following, to discuss SERS response, we use the intensity values of the most pronounced NBT Raman band at 1343 cm^−1^. The coating of nanoparticles with a silver shell leads to a significant increase in the SERS signal from molecules on the surface of a gold nanorod. This may be due to the well-known fact that silver is a better Raman enhancer than gold due to smaller resistive losses [39]. The SERS spectra measured from colloids after coating the nanoparticles with a silver shell are also shown in Figure 3a. It can be seen that, for gold nanorods, the maximum SERS signal was about 800 counts. Coating with a silver shell in the Ag/Au molar ratio of 1.25 led to an increase in the signal up to 27,000 counts. For triangular pyramids, a maximum signal of about 40,000 counts was observed. At the maximum ratio of silver to gold, the signal was somewhat smaller and reached 24,000 counts. Interestingly, their SERS response did not exhibit a monotonic increase during Ag shell formation. It was found that the SERS response of nanoparticles increased with the increase in the Ag/Au ratio from 0 to 2.5 but started to decrease with more amount of Ag (Figure 3b). This is because a too-thick Ag shell may hinder light penetration and counteract the Raman enhancement [40].

Thus, we synthesized three types of Au@Ag nanorods with incorporated Raman reporters. The nanoparticles differed in morphology (core–shell nanorods, triangle pyramid/octadecahedron), plasmon resonance shift, and exhibit enhanced SERS, compared with uncoated AuNRs. Further, these nanoparticles were used for the detection of hydrogen peroxide by the method of controlled Ag shell etching.

### 3.2. Colorimetric Detection of H_2_O_2_

The main color and spectral changes that occurred in colloids of Au@Ag nanoparticles upon the addition of hydrogen peroxide are based on the following reaction:

2Ag + H_2_O_2_ → 2Ag^+^ + 2OH (1)

Depending on the amount of hydrogen peroxide added, partial or complete dissolution of the silver shell should be observed. This leads to the reverse color and spectral changes observed during shell growth.

**Figure 4:**
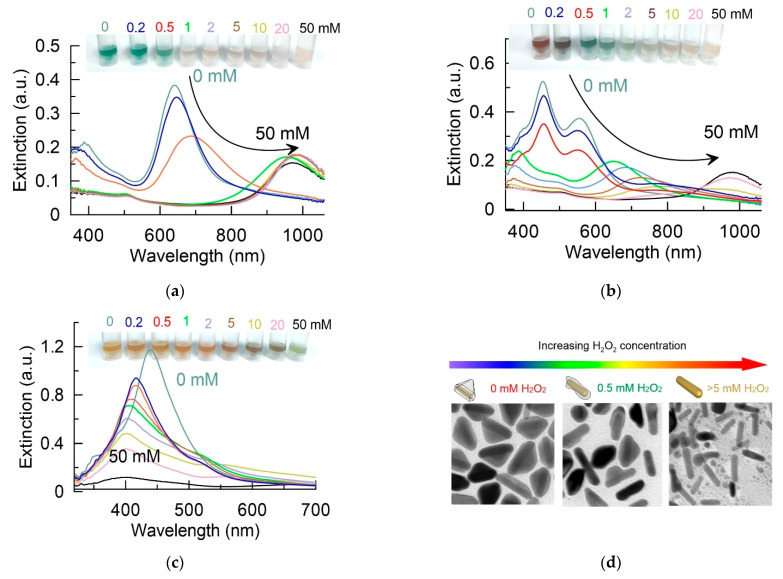
The change in Au@Ag nanoparticles extinction spectra as a function of the concentration of H_2_O_2_. The data are given for nanoparticles obtained using Ag-to-Au molar ratios 1.25 (**a**), 2.5 (**b**), and 5 (**c**). The insert shows images with nanoparticles colloids; (**d**) representative TEM images of Au@Ag nanoparticles before and after addition of H_2_O_2_ to final concentrations of 0.5 and 5 mM.

Figure 4a–c shows the changes in extinction spectra for Au@Ag nanoparticles after incubation in H_2_O_2_ at different concentrations. The insert shows color changes in nanoparticle colloids. It should be noted that, for the three types of colloids under study, there were differences in spectral and color changes. Let us first discuss the measurement results for nanorods obtained using the minimum amount of silver (Figure 4a). Upon incubation with hydrogen peroxide at a concentration of up to 0.5 mM, a decrease in the optical density of the colloid and a shift in the plasmon resonance to the long-wavelength region were observed. This may indicate a partial dissolution of the silver shell. With a further increase in the concentration of hydrogen peroxide to 1 mM or more, a dramatic change in the optical properties of the colloid occurred. The shift in the resonance to the region of 900–920 nm indicated the complete dissolution of the silver shell. The color of the colloid also changed dramatically from the original green to brown-orange. Such a sharp concentration-dependent transition indicated the autocatalytic nature of the reaction of hydrogen oxide with silver in the composition of the shell [41].

For the case of a thicker silver shell (Figure 4b), the color and spectral changes differed from those obtained for the first sample. With an increase in the concentration of hydrogen peroxide from 0 to 0.5 mM, a decrease in the optical density of the colloid and a slight short-wave shift in the plasmon peak from 550 to 540 nm were observed. We assume that these changes are due to the rounding of the sharp edges of the triangular pyramid. It is well known that metal facets exhibit a higher chemical reactivity, compared with a bulk sample [42]. A further increase in the concentration of hydrogen peroxide from 0.5 to 5 mM led to a long-wavelength shift in the plasmon resonance from 540 to 800 nm, depending on the concentration of H_2_O_2_. We unequivocally interpreted these changes as a progressive dissolution of the silver shell. Finally, at high concentrations of hydrogen peroxide (20–50 mM), the silver shell completely dissolved, and the extinction spectrum corresponded to gold nanorods. Our assumptions were confirmed by the data of TEM measurements (Figure 3d). Without the addition of H_2_O_2_, the particles mostly had a triangular pyramid shape. After incubation with hydrogen peroxide at a concentration of 0.5 mM, the shell was partially dissolved, and the faces were smoothed. The addition of peroxide at a concentration of 5 mM or more led to the complete dissolution of the silver shell. The spectral changes discussed above led to a sequential change in the color of the colloid from red to blue, green, yellow-green, and brown depending on the concentration of added hydrogen peroxide.

Let us now consider the case of Au@Ag nanoparticles obtained using the maximum concentration of silver. Etching of the silver shell in this case led to a drop in the optical density of the colloid and a short-wavelength shift in the plasmon resonance (Figure 4c). Only when using the highest concentrations of hydrogen peroxide, a long-wavelength peak was observed in the spectrum, which is responsible for the extinction of the gold nanorod. The color of the colloid remained yellow over a wide concentration range up to 20 mM. Additionally, only at the maximum concentration of H_2_O_2_ did the color change to green due to the appearance of the second plasmon peak.

To summarize the data of spectral measurements, we can conclude that we did not find a single parameter (decrease in optical density, plasmon shift, etc.) that can be used to construct a calibration curve for determining the concentration of hydrogen peroxide. Indeed, for a thin gold shell, a sharp transition in the spectrum was observed, with the complete disappearance of the shell. At average values of the amount of silver, the predominant effect was the plasmon shift. At the maximum amount of silver, a decrease in optical density was observed, without a significant plasmon shift. Therefore, we further decided to turn to the study of changes in the SERS properties of Au@Ag nanoparticles upon etching of the silver shell.

### 3.3. SERS Detection of H_2_O_2_

In contrast to measurements of extinction spectra, where it is necessary to take into account both the shift in the plasmon peak and the drop in its intensity, SERS allows using only one parameter characterizing the dissolution reaction of the silver shell. The principle of SERS detection is as follows: The investigated solution of hydrogen peroxide is added to the Au@Ag colloid with incorporated Raman reporters. During the reaction with hydrogen peroxide, the silver shell dissolves, which leads to a decrease in SERS from Raman molecules. By measuring the Raman spectra before and after etching, it is possible to construct a calibration curve for the dependence of the SERS intensity on the H_2_O_2_ concentration. Figure 4a shows SERS spectra obtained by incubating Au@Ag triangular pyramids (Ag/Au = 2.5) with hydrogen peroxide at concentrations from 0.1 to 50 mM. In all cases, the spectral signature corresponded to NBT. This indicated the absence of chemical modification of the repeater molecules. As expected, there was a progressive decrease in the intensity of the SERS signal with an increase in the concentration of hydrogen peroxide. For initial Au@Ag particles, the intensity of the SERS line of the nitro group vibration (1343 cm^−1^) was about 40,000 counts. Complete etching of the silver shell reduced this signal to a level corresponding to gold nanorods, equal to 800 counts. To quantify the dependence of the SERS level on the concentration of hydrogen peroxide, we proposed to use the parameter relative intensity loss (RIL).

(2) $$\mathrm{RIL}\;=\;\frac{I_{\mathrm{etched}}^{\mathrm{SERS}}{\;-\;I}_{\mathrm{nanorods}}^{\mathrm{SERS}}}{I_{\mathrm{initial}}^{\mathrm{SERS}}}$$

This parameter is determined by Equation (2) and shows how much the SERS intensity decreases in relative terms, compared with the boundary values (initial particles before etching of the silver shell, gold nanorods).

**Figure 5:**
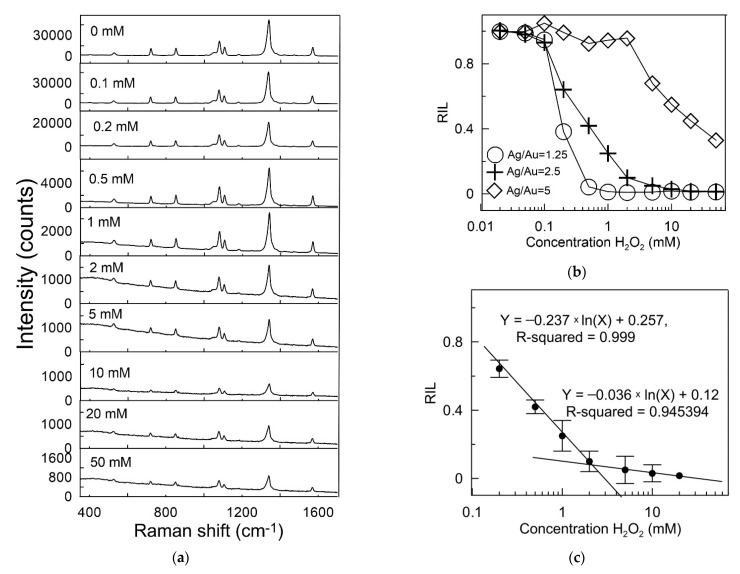
(**a**) The Au@Ag nanoparticles’ SERS spectra were measured after incubation with H_2_O_2_ at different concentrations. The data are given for nanoparticles obtained using Ag-to-Au molar ratio of 2.5; (**b**) the dependence of relative intensity loss (RIL) on the concentration of H_2_O_2_; (**c**) two linear calibration curves by plotting the RIL versus different concentrations of H_2_O_2_. within 0.1–2 mM and 2–20 mM.

Obviously, for the case of complete dissolution of the silver shell, RIL is equal to 0, and for the initial particles, it is close to 1. Therefore, this universal parameter makes it possible to compare the efficiency of the SERS reaction of different particles to hydrogen peroxide. Figure 5b shows the dependence of RIL on the concentration of hydrogen peroxide for three types of particles under study. In relative agreement with the colorimetric data for nanoparticles with a thin silver shell (shown by circles), RIL was about 1 for concentrations up to 0.2 mM. Further, a sharp decrease in RIL was observed, and already at a concentration of 0.5 mM, it became close to zero. This means that the limit of detection (LOD) was 0.2 mM, and the working range was 0.2–0.5 mM. The reverse situation was observed for nanoparticles with the maximum thickness of the silver shell (shown by rhombs). The change in RIL began only with a hydrogen peroxide concentration of 2 mM. However, RIL did not drop to 0 even for the maximum concentration of H_2_O_2_. When using triangular pyramids (shown as crosses), the widest working range of 0.1–20 mM was observed. Therefore, we chose this sample to build a calibration curve for determining the concentration of H_2_O_2_ from the change in SERS. Figure 5c shows the relationship between RIL and the logarithmic value of H_2_O_2_ concentration. Two linear calibration curves were obtained for hydrogen peroxide concentration range 0.2–2 mM and 2–20 mM, with a limit of detection of 0.1 mM, and the regression equation were RIL = −0.237ln (H_2_O_2_ in mM) + 0.257 (R = 0.999) and RIL = −0.036ln (H_2_O_2_ in mM) + 0.12 (R = 0.945), respectively. It should be noted that the two logarithmic ranges of the calibration curve were typical for hydrogen peroxide sensing based on the dissolution of silver particles [22]. We believe that this is due to the self-acceleration of the reaction due to its autocatalytic nature. It should be noted that the detection limit of sensors based on silver nanoparticles can be significantly reduced in the future (by two or three orders of magnitude) due to the rational design of the surface ligand [18] or doping of nanoparticles with iodine atoms [43].

### 3.4. Anti-Interference and Stability of the Sensing

Various parameters can affect the efficiency of the determination of hydrogen peroxide, reducing its effectiveness. We first decided to test the effect of pH on the selective oxidation of the silver shell. Hydrochloric acid and sodium hydroxide were used to adjust the pH of the H_2_O_2_ solution, which was then tested using prepared Au@Ag nanoparticles. In general, we did not find a significant difference in the rate and magnitude of SERS when incubating nanoparticles in solutions of pH 3.6 and 9. This result shows that our enzyme-free method overcomes the difficulties of bioenzymatic pH sensitivity.

We also evaluated H_2_O_2_ recovery in five samples by comparing detection results with actual concentrations (Table 1). Detection recovery was distributed from 90% to 116%, relative standard deviation from 3% to 12%, indicating that the proposed sensing has a good potential for use in practical applications. To evaluate the specificity of the SERS sensing, interference experiments were carried out by using different organic and inorganic substances. As we are not comparing different types of particles here, we simply used the decrease in SERS signal as a quantitative characteristic. The SERS signal intensities of NBT at 1343 cm^−1^ were individually measured in samples treated with sodium chloride, glucose, vitamin C, methanol, nickel ions, dopamine, l-arginine, or a mixture containing H_2_O_2_ and the above-mentioned interference substances at the same concentration of 10 mM. As shown in Figure S3 (Supporting information), none of the reagents by themselves led to a significant drop in SERS. Thus, we can conclude that discussed reagents did not promote silver shell etching. However, with the further addition of H_2_O_2,_ the SERS signal from nanoparticles decreased significantly, compared with the control signal. The exception here is the progressive addition of ascorbic acid and H_2_O_2_ to the Au@Ag nanoparticles. In this case, there was no considerable change in the SERS signal both after ascorbic acid addition and after further H_2_O_2_ addition. We believe that it is because of the well-known antioxidant properties of vitamin C [44].

Despite the wide operating range and the absence of serious interference with other biomolecules, the proposed SERS-based sensing has several disadvantages that limit its use in some areas, especially in the point-of-care detection of H_2_O_2_. First, the SERS format for reading the result involves the use of special equipment and signal processing techniques. Second, because the test is based on an etching process, the required time for analysis is several hours. Finally, as shown above, the sensitivity of the method strongly depends on the type of used core–shell nanoparticles. Therefore, quality control and calibration are necessary steps for each new synthesis of nanoparticles.

### 3.5. Indicator Paper for H_2_O_2_ Sensing

The colorimetric and SERS methods discussed above for determining the concentration of hydrogen peroxide are based on experiments in a colloid. Additionally, the experiment requires specialized equipment and cannot be considered point of care. On the other hand, there are some tasks, for example, the determination of the content of peroxides in food, for which the simplicity and efficiency of the technique are of paramount importance.

We noted above that the incubation of nanoparticles with different concentrations of peroxide led to color changes in the colloid. Using this fact, we decided to develop the simplest indicator paper for H_2_O_2_ point-of-care detection.

**Figure 6:**
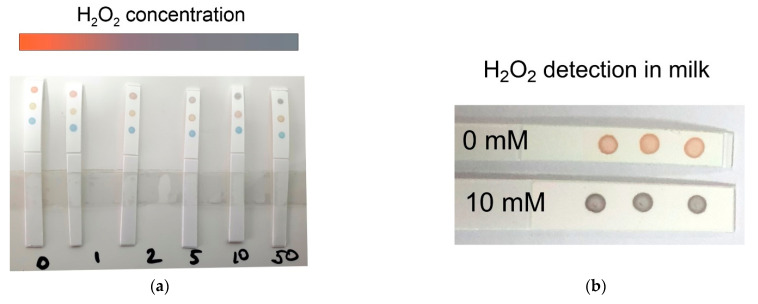
(**a**) Color change in nanoparticles deposited on a nitrocellulose membrane upon incubation with hydrogen peroxide at a concentration of 0–50 mM. Nanoparticles were obtained using Ag-to-Au molar ratios of 2.5 (upper dots), 5 (middle dots), and 1.25 (bottom dots). The color decoding bar is shown on the top; (**b**) an example of measurement of H_2_O_2_ in the spiked milk sample.

To this end, we applied the nanoparticles to the nitrocellulose membrane in the form of dots (Figure 6). It should be noted that when dried, the samples with a high Ag/Au molar ratio did not change color (red and yellow dots). A sample with a molar ratio of silver to gold of 1.25 changed color from green to blue-green. We then incubated the membrane in a hydrogen peroxide solution with predetermined concentrations from 0 to 50 mM for 1 min. Next, the membrane was dried, and the change in the color of the spots was analyzed. The largest color changes, as expected, were observed for a sample with a silver/gold molar ratio of 2.5. Thus, at concentrations of 0–1 mM, the color of the spot remained red, 2–5 mM gray-red, and more than 5 mM gray. We compiled a color decoding map of the dependence of the spot color on the concentration of peroxide (Figure 6a). To test the applicability of our indicator paper to the analysis of real samples, we measured the concentration of peroxide in a contaminated milk sample. As can be seen from Figure 6b, the color changes in triplicate are fully consistent with those expected according to the color map and the added concentration of peroxide. The proposed indicator paper had an order of magnitude lower sensitivity, compared with SERS sensing. On the other hand, it did not require any special equipment, and the analysis took only 10 min. To the best of our knowledge, this is the first demonstration of hydrogen peroxide detection that can be used by point of care. Thus, we developed an indicator paper for the rapid assessment of food contamination with hydrogen peroxide.

## 4. Conclusions

In summary, we developed a nonenzymatic H_2_O_2_ sensor with the advantages of simple operation, fast response, and excellent selectivity. The sensing was based on the selective etching of Au@Ag nanorods with embedded Raman active molecules. The readout of the signal was performed by changing the color of the particles, the shift, and decrease in the plasmon peak, and/or the decrease in the SERS signal from the embedded Raman molecules. We found that core–shell nanoparticles with a Ag/Au molar ratio of 2.5 exhibited the highest sensitivity, with a detection limit of 10^−4^ M, and a SERS detection range of 1 × 10^−4^ to 2 × 10^−2^ M. When applied on nitrocellulose paper, these nanoparticles can serve as simple indicator paper sensor for fast detection of hydrogen peroxide in liquids. Therefore, we believe that the proposed method has a broad prospect in food safety, industry, and environmental monitoring of H_2_O_2_.

## Figures and Tables

**Table 1:** H_2_O_2_ recovery tested by using the SERS sensor.

Sample	Added, mM	Found, mM	Recovery (%)	Standard Deviation (*n* = 3), %
1	0.5	0.45	90%	4%
2	2	2.15	107%	5%
3	2.5	2.7	108%	3%
4	3	3.5	115%	12%
5	5	5.5	110%	6%

## Supplementary Materials

The following are available online at https://www.mdpi.com/article/10.3390/s22093202/s1. Figure S1: Extinction spectra of AuNRs. The insert shows a typical TEM image of the nanorods, Figure S2: Color changes during the growth of a silver shell on the surface of a gold nanorod at various Ag/Au molar ratios, Figure S3: Possible interferences tested with the H_2_O_2_ sensor: (a) 10 mM H_2_O_2_, sodium chloride, ascorbic acid, glucose, methanol, nickel nitrate, dopamine, l-arginine; (b) adding another10 mM H_2_O_2_ in the above solutions, Table S1 Comparison of the sensitivity and speed of various methods for the determination of hydrogen peroxide.
