# Supplementary material for: SERS and Indicator Paper Sensing of Hydrogen Peroxide Using Au@Ag Nanorods
Source: Sensors (Basel). 2022 Apr 21;22(9):3202. doi: 10.3390/s22093202 (PMC9101113; doi:10.3390/s22093202)
Supplement: Supplementary file 1 [file sensors-22-03202-s001.zip › sensors-1654902-supplementary.pdf]

Supplementary Materials

SERS and Indicator Paper Sensing of Hydrogen Peroxide Using Au@Ag Nanorods

Boris N. Khlebtsov [1,*], Andrey M. Burov [1], Andrey M. Zakharevich [2] and Nikolai G. Khlebtsov [1,2]

[1] Institute of Biochemistry and Physiology of Plants and Microorganisms, Saratov Scientific Centre of the Russian Academy of Sciences (IBPPM RAS), 410049 Saratov, Russia; burov_a@ibppm.ru (A.M.B.); khlebtsov_n@ibppm.ru (N.G.K.)

[2] Science Medical Center, Saratov State University, 410012 Saratov, Russia; lab-15@mail.ru

* Correspondence: khlebtsov_b@ibppm.ru; Tel.: +7-8452-97-0403

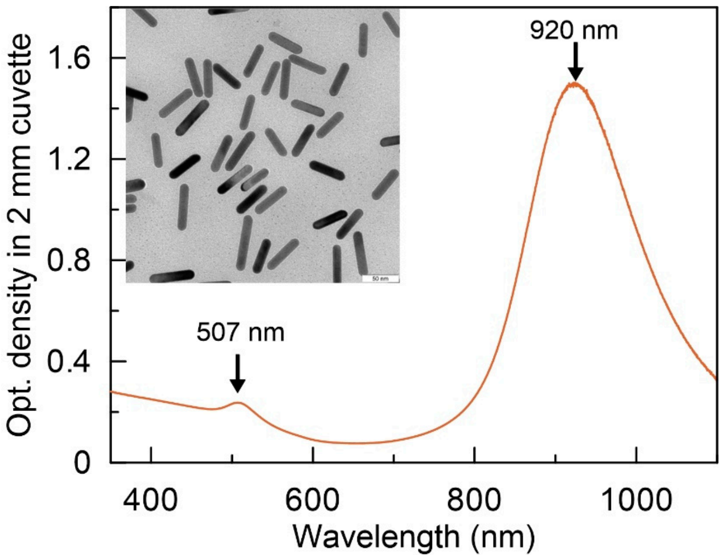

Figure S1. Extinction spectra of AuNRs. The insert shows a typical TEM image of the nanorods.

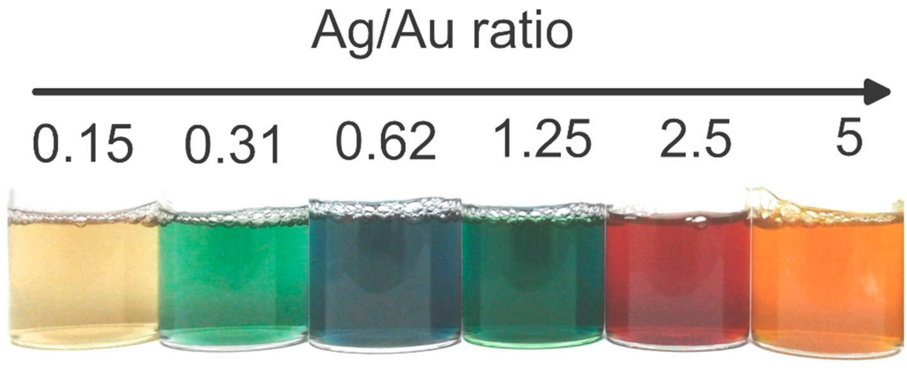

Figure S2. Color changes during the growth of a silver shell on the surface of a gold nanorod at various Ag/Au molar ratios.

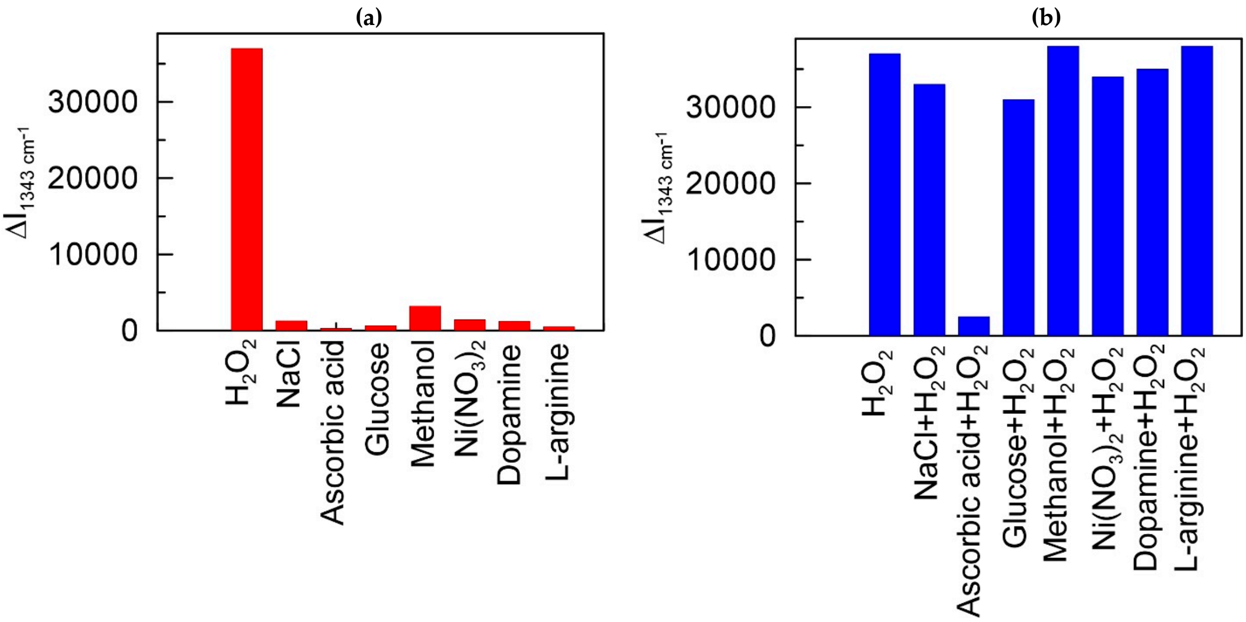

Figure S3. Possible interferences tested with the H_2O_2sensor: (**a**) 10 mM H_2O_2, sodium chloride, ascorbic acid, glucose, methanol, nickel nitrate, dopamine, l-arginine; (**b**) adding another 10 mM H_2O_2 in the above solutions.

Table S1. Comparison of the sensitivity and speed of various methods for the determination of hydrogen peroxide.

Nanoparticle type	Detection method	Detection range	Time for assay	Reference
Biogenic silver nanoparticles	Colorimetric	1-10 mM	1 hour	[20]
Ag nanospheres	Colorimetric	0.1-50 mM	1 hour	[42]
Siver/carbon nanomaterial	Colorimentric	0.01-0.2 mM	30 min	[19]
Ag nanocubes<br>Ag nanospheres<br>Ag prisms	Colorimentric	1-10 mM<br>0.1-10 mM<br>0.02-10 mM	2 hour	[24]
Fluorescent/Ag microparticles	Fluorescence	0.001-0.5 mM	1 hour	[17]
Ag NP-assembled substrate	SERS	0.01-0.1 mM	2 hour	[26]
Au@Ag nanospheres	SERS	0.01-0.3 M	2 hour	[31]
Au@Ag nanorods	SERS	0.1-20 mM	1 hour	This paper
Indicator paper	Naked eye	1-50 mM	10 min	This paper

References

17. Lu, H.; Yu, C.; Quan, S.; Xu, S. A novel dual response ratiometric fluorescent probe for the determination of H_2O_2 and glucose via etching of silver nanoparticles. *Analyst* **2019**, *144*, 1153–1158.
19. Hou, W.; Liu, X.; Lu, Q.; Liu, M.; Zhang, Y.; Yao, Sh. Etching and anti–etching strategy for sensitive colorimetric sensing of H2O2 and biothiols based on silver/carbon nanomaterial. *Colloids Surf. B Biointerfaces* **2018**, *162*, 118–125.
20. Roy, K.; Sarkar, Ch.K.; Ghosh, Ch.K. Fast colourimetric detection of H_2O_2 by biogenic silver nanoparticles synthesised using *Benincasa hispida* fruit extract. *Nanotechnol. Rev*. **2016**, *5*, 251–258.
26. Li, Y.; Wang, Y.; Fu, C.; Wu, Y.; Cao, H.; Shia, W.; Jung, Y.M. A simple enzyme-free SERS sensor for the rapid and sensitive detection of hydrogen peroxide in food. *Analyst* **2020**, *145*, 607–612.
24. Zhang, L.; Li, L. Colorimetric detection of hydrogen peroxide using silver nanoparticles with three different morphologies. *Anal. Meth.* **2016**, *8*, 6691–6695.

31. Wang, X.; Zhang, K.; Tan, X.; Zhang, Y.; Bai, L.; Xie, W. Etchable SERS nanosensor for accurate pH and hydrogen peroxide sensing in living cells *Chem. Commun.* **2019**, *55*, 12996–12999.
42. Meng, F.; Zhu, X.; Miao, P. Study of autocatalytic oxidation reaction of silver nanoparticles and the application for nonenzymatic H_2O_2 assay. *Chem. Phys. Lett.* **2015**, *635*, 213–216.
